# Kinetics of neurodegeneration based on a risk-related biomarker in animal model of glaucoma

**DOI:** 10.1186/1750-1326-8-4

**Published:** 2013-01-18

**Authors:** Takuya Hayashi, Masamitsu Shimazawa, Hiroshi Watabe, Takayuki Ose, Yuta Inokuchi, Yasushi Ito, Hajime Yamanaka, Shin-ichi Urayama, Yasuyoshi Watanabe, Hideaki Hara, Hirotaka Onoe

**Affiliations:** 1 Functional Probe Research Laboratory, RIKEN Center for Molecular Imaging Science, Kobe, Hyogo, 650-0047, Japan; 2 Department of Investigative Radiology, National Cerebral and Cardiovascular Center Research Institute, Suita, Osaka, 565-8565, Japan; 3 Human Brain Research Center, Kyoto University Graduate School of Medicine, Kyoto, 606-8507, Japan; 4 Center for iPS cell Research and Application, Kyoto University, Kyoto, 606-8507, Japan; 5 Department of Physiology, Osaka City University Graduate School of Medicine, Osaka, 545-8586, Japan; 6 Molecular Pharmacology, Department of Biofunctional Evaluation, Gifu Pharmaceutical University, Gifu, 501-1196, Japan; 7 Faculty of Molecular Imaging in Medicine, Osaka University Graduate School of Medicine, Osaka, 565-0871, Japan; 8 Molecular Probe Dynamics Laboratory, RIKEN Center for Molecular Imaging Science, Kobe, Hyogo, 650-0047, Japan

**Keywords:** Diffusion tensor, Neurodegenerative mechanisms, Biomarker, Kinetic model, Glaucoma

## Abstract

**Background:**

Neurodegenerative diseases including Parkinson’s and Alzheimer’s diseases progress slowly and steadily over years or decades. They show significant between-subject variation in progress and clinical symptoms, which makes it difficult to predict the course of long-term disease progression with or without treatments. Recent technical advances in biomarkers have facilitated earlier, preclinical diagnoses of neurodegeneration by measuring or imaging molecules linked to pathogenesis. However, there is no established “biomarker model” by which one can quantitatively predict the progress of neurodegeneration. Here, we show predictability of a model with risk-based kinetics of neurodegeneration, whereby neurodegeneration proceeds as probabilistic events depending on the risk.

**Results:**

We used five experimental glaucomatous animals, known for causality between the increased intraocular pressure (IOP) and neurodegeneration of visual pathways, and repeatedly measured IOP as well as white matter integrity by diffusion tensor imaging (DTI) as a biomarker of axonal degeneration. The IOP in the glaucomatous eye was significantly increased than in normal and was varied across time and animals; thus we tested whether this measurement is useful to predict kinetics of the integrity. Among four kinds of models of neurodegeneration, constant-rate, constant-risk, variable-risk and heterogeneity models, goodness of fit of the model and *F*-test for model selection showed that the time course of optic nerve integrity was best explained by the variable-risk model, wherein neurodegeneration kinetics is expressed in an exponential function across cumulative risk based on measured IOP. The heterogeneity model with stretched exponential decay function also fit well to the data, but without statistical superiority to the variable-risk model. The variable-risk model also predicted the number of viable axons in the optic nerve, as assessed by immunohistochemistry, which was also confirmed to be correlated with the pre-mortem integrity of the optic nerve. In addition, the variable-risk model identified the disintegrity in the higher-order visual pathways, known to underlie the transsynaptic degeneration in this disease.

**Conclusions:**

These findings indicate that the variable-risk model, using a risk-related biomarker, could predict the spatiotemporal progression of neurodegeneration. This model, virtually equivalent to survival analysis, may allow us to estimate possible effect of neuroprotection in delaying progress of neurodegeneration.

## Background

Neurodegenerative diseases are slowly progressive and intractable disorders of the nervous system. The temporal profile of neurodegenerative diseases shows a pattern of high variability across patients in terms of symptoms, neuropathology and neuroimaging findings [1-3]. This variability, as well as their slowly progressive nature, poses difficulty in assessing treatment efficacy [3]. For any current and future drug that potentially delays disease progression, it is important to know whether and how much it delays deterioration caused by neurodegenerative diseases. Early detection of disease is now becoming possible by measurement of neurochemical [4,5] and neuroimaging [6] biomarkers specifically related to the pathogenic events (for review, see [7,8]. Although several biomarker models have been proposed to illustrate the relationship between the biomarker cascade and symptomatic/clinical stages [1,2], there is scarce evidence that biomarkers can be used to quantitatively interpret the neurodegenerative process. To this end, it is necessary to establish a model for the kinetics of neurodegeneration using a biomarker, and to predict the time courses for testing the long-term efficacy of treatment.

**Figure 1:**
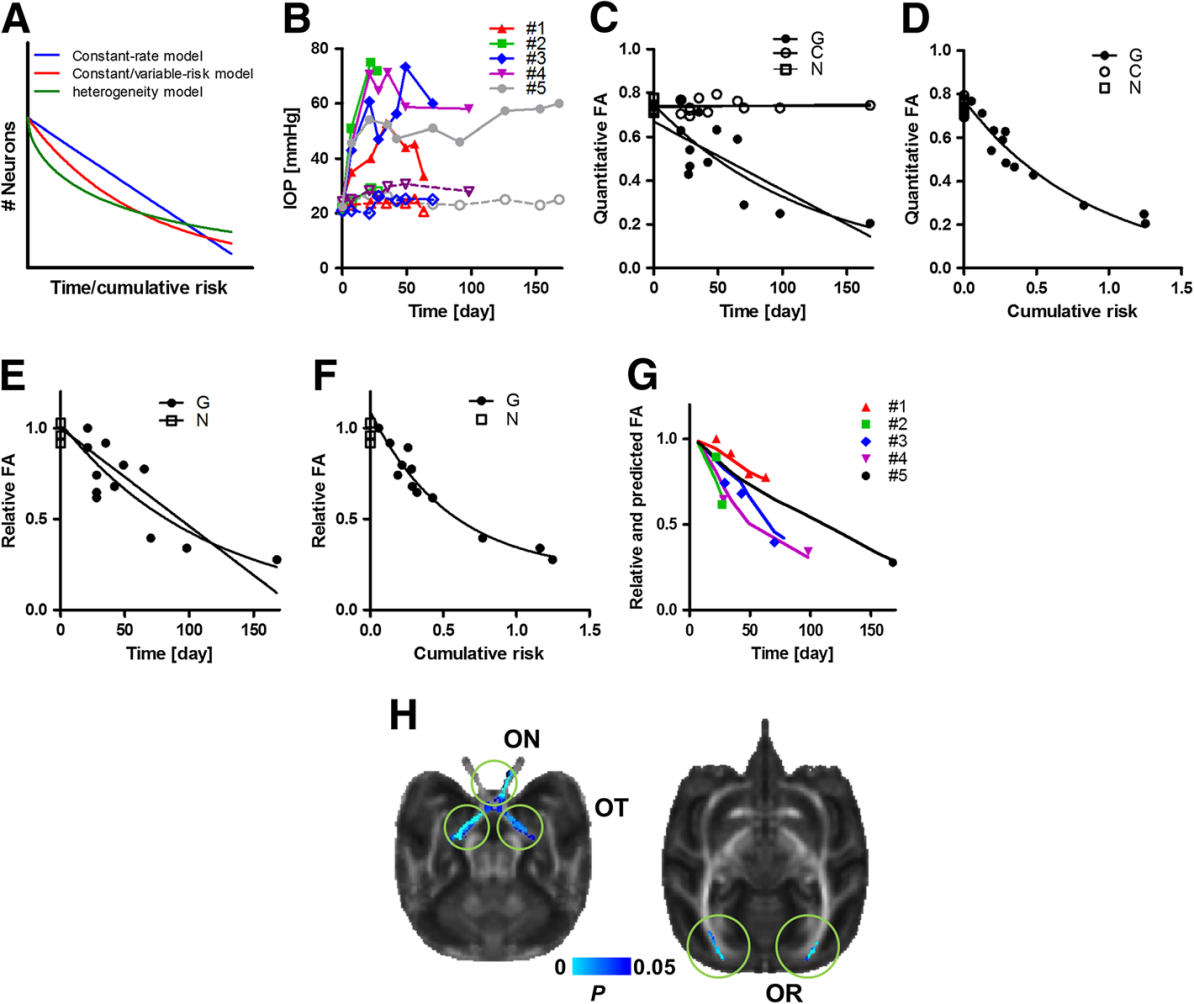
**Kinetic model of neurodegeneration, time course of risk (intraocular pressure) and MRI-based neurodegeneration in glaucomatous animals. (A)** Four putative kinetic models of neurodegeneration: 1) constant-rate model (blue straight line with x-axis of time after the onset of disease), 2) constant-risk model (red exponential line with x-axis of time), 3) variable-risk model (red exponential line with x-axis of cumulative risk), and 4) heterogeneity (+variable risk) model (green stretched exponential decay line with x-axis of cumulative risk). (**B**) Time courses of intraocular pressure (IOP) in animals with glaucoma. Closed point and thick line, the glaucomatous eye (**G**); open point and dashed line, control (contralateral side) eye. (**C**). The shapes of the points specify each of the animals: animal #1 (▲△); #2 (∎□); #3 (⋄♦); #4 (▼∇); and #5 (●○). (**C**) Time course of quantitative values of fractional anisotropy (FA) in the optic nerves of glaucomatous eye (G-ON), control (C-ON, contralateral side), and in those in normal animals (N-ON). Constant-rate model and constant-risk model are also fit to the G-ON data. See also Table 1. (**D**) A plot of quantitative FA values (in y-axis) across cumulative risk (*∫r(t)dt,* in Eq. 2) in the variable-risk model (solid line). (**E**) Time course of relative FA values (rFA) in the glaucomatous optic nerve expressed as a ratio to the control optic nerve (**G**) and a ratio, left-to-right, in normal animals (N). Constant-rate and constant-risk models are also fit to the ratio of G. (**F**) A plot of rFA values in the glaucomatous optic nerve (y-axis) and cumulative risk (x-axis) in glaucomatous animals (**G**) and normal animals (N). The glaucomatous FA values are fit by the variable-risk model (solid line: fitted curve). (**G**) A plot of predicted neurodegeneration (line) and actual rFA values (points) in each animal of #1-5. The prediction of rFA were calculated based on the initial value (*N*_*0*_ =1) and optimized values (*r*_*0*_ = 0.0019, *β* = 0.057) in the variable-risk model. (**H**) Results of the voxel-based analysis of FA images with cumulative IOP as a regressor. A significant cluster (*P* < 0.05, corrected for multiple comparison) is shown in blue. ON, optic nerve; OT, optic tract; and OR, optic radiation. See Additional file 2: Table S2 for a list of all significant regions.

Classical prediction models kinetics of neurodegeneration simply in the context of accelerated aging [9], i.e., once initiated, neurodegeneration proceeds at a rate faster than in normal-aging [10] (constant-rate model in Figure 1A), but this model ignores the causality of disease-specific risk. A more elaborate interpretation has its basis in a risk-based stochastic model, in which neurodegeneration occurs as probabilistic events for each neuron depending on the risk. If the risk is time-invariable, the number of neurons should decrease as an exponential function of time, like the radioactivity decay of a radioisotope (a constant-risk model in Figure 1A). This was shown to be the case *in vitro*[11,12], and in cases of hereditary neurodegeneration [11,13]. Although this model reasonably considers neurodegeneration as probabilistic events, it oversimplifies the situation of neurodegeneration in the brain. For example, the risk is not always constant in most types of non-inherited neurodegenerative diseases, as is made evident by the between-subject variability in symptoms or neuropathologies [1-3]. In addition, interactions between neurons seem to underlie transsynaptic degeneration in the secondary neurons which have synaptic connections with primarily dying neurons [14-16]. Thus neurodegeneration is not a series of independent events like radioisotope decay. Therefore, the stochastic model needs to be qualified when neurons are exposed to a variable level of risks and/or transsynaptic death. We modeled a condition where the level of risk is time-variable (see Kinetic models of neurodegeneration in the Methods section), and thereby predicted that the number of neurons should decrease exponentially with time-integrated risk, i.e. cumulative risk (variable-risk model in Figure 1A). We also modeled transsynaptic death, the kinetics of which are linear to the primary neuronal death, thus allowing detection of the distribution of neurodegeneration across the brain.

Glaucoma, a leading cause of the adult-onset blindness, is known to involve a pathology in which axons of retinal ganglion neurons are mechanically injured by a chronic increase in intra-ocular pressure resulting from dysregulated aqueous fluid circulation [17]. In this disease, neurodegeneration occurs not only in the retinal ganglion neuronal cell bodies and their axons in the optic nerves/tracts, but also transsynaptically in the lateral geniculate nucleus, optic radiation, and visual cortex [14-16]. The causality between increased IOP and glaucomatous neurodegeneration is well established based on several observations: 1) the higher the IOP, the more accelerated is the progression of glaucoma [18]; 2) therapies that alleviate IOP prevent the progress of the disease [18]; and 3) experimental animals given a treatment that increases IOP by blocking aqueous-fluid absorption exhibit typical visual-field deficits and retinal pathologies similar to human patients [19,20]. Although its primary cause is different, the glaucoma shares close similarities with Parkinson’s and Alzheimer’s disease in several pathological findings [21] and is recently considered as one of neurodegenerative diseases that need neuroprotective therapies [22].

Here, using diffusion-weighted magnetic resonance imaging, we test whether the causal model explains the time course of neurodegeneration in the living brains of experimental animals with an established neurodegeneration model. The technique of diffusion-weighted magnetic resonance imaging has recently enabled *in vivo* measurements of white matter microstructure, thus allowing the quantitative, longitudinal assessment of neurodegeneration in various diseases including Alzheimer’s [23,24] and Parkinson’s diseases [25,26], amyotrophic lateral sclerosis [27,28] and glaucoma [29]. By applying the diffusion tensor model [30], diffusion-weighted data could be used to calculate fractional anisotropy (FA), a scale that expresses anisotropic diffusion motion and is proven to be correlated with the density of viable neuronal axons if conditions permit (for review, see [31]). We used macaque monkeys (*Macaca fascicularis*) with glaucoma induced by laser photocoagulation of the trabecular meshwork [32], which is known to result in a chronic increase in IOP and a pathology that mimics glaucoma, degeneration of axons of retinal ganglion neurons.

## Results

The animals with glaucoma showed increased IOP in the affected eye (Figure 1B and Additional file 1: Table S1) during the follow-up period from 33 to 168 days after laser coagulation that blocks aqueous fluid absorption. The mean IOP values in the glaucomatous eye was higher than those in the baseline (54.9 ± 4.2 vs. 23.2 ± 0.8 mmHg, paired-*T* test, *P* < 0.005) or than the contralateral (22.8 ± 0.73, *T* test, *P* < 0.05, Additional file 1: Table S1). In addition, as has been seen in our previous study [20], values of IOP in the glaucomatous eye were significantly variable across time (analysis of covariance, *F*_*17,19*_ = 4.34, *P* < 0.05) and subject (*F*_*1,19*_ = 5.35, *P* < 0.05) (see Figure 1B), which led us to assume that the variability in IOP fluctuates with the rate of progression of degeneration.

The FA values in the affected optic nerve were also variable across time (*F*_*1,6*_ = 20.0, *P* < 0.005) and subjects (*F*_*4,6*_ = 9.59), *P* < 0.01), whereas not those of the non-affected optic nerve (Figure 1C). Besides time course of quantitative FA values, we also used those of relative FA values in the subsequent analysis of model fitting with the view to reduce effects of measurement error. The relative value of FA was expressed as a ratio of values for the affected optic nerve to those for the non-affected nerve. When we fitted the constant-rate model to the FA and time, the variance of FA was only slightly explained by time. A coefficient of determination (*R*^*2*^) was 0.35 for quantitative FA (linear regression analysis, *P* < 0.05, Figure 1C) and 0.36 for relative FA values (*P* < 1.0 × 10^-4^, Figure 1E, see also Table 1). The FA values were better fitted by the stochastic constant-risk model (*R*^*2*^ = 0.67, *P* < 1.0 × 10^-3^, Figure 1C and *R*^*2*^ = 0.89, *P* < 1.0 × 10^-5^, Figure 1D, for quantitative and relative FA data, respectively, see also Table 1). The FA data were much better fitted by the stochastic variable-risk model, based upon the cumulative risk that was assumed to be proportional to the power of the IOP (quantitative FA: *R*^*2*^ = 0.94, *P* < 1.0 × 10^-6^, Figure 1D, relative FA *R*^*2*^ = 0.98, *P* < 1.0 × 10^-7^, Figure 1F, Table 1). An *F*-test for model selection disclosed that the variable-risk model explained the data significantly better than the constant-risk (quantitative FA: *F*_*1,9*_ = 38.6, *P* < 0.005; relative FA: *F*_*1,10*_ = 37.9, *P* < 0.0005, Table 1). We also tested a model recently proposed as one that explains kinetics of neurodegeneration with heterogeneity using a stretched exponential decay function [33] (see section of Kinetic model of neurodegeneration in Methods and Figure 1A), which is often used in the field of physics to describe the relaxation in disordered system [34]. The heterogeneity model well fit to both of the quantitative and relative FA data (*R*^*2*^ = 0.96, *P* < 1.0 × 10^-7^ and *R*^*2*^ = 0.98, *P* < 1.0 × 10^-8^, respectively, Table 1), and an optimized value of parameter, γ was less than 1 for both of quantitative and relative FA values (0.58 and 0.98, respectively, Table 1) as expected in a typical stretched exponential decay model (see Methods). However, an *F*-test for model selection showed that the heterogeneity model did not significantly improve explanation of data variability as compared with the variable-risk model (*F*_*1,8*_ = 3.5; *F*_*1,9*_ = 0.6, Table 1).

**Table 1:** Parameter estimates in the constant-rate and risk-based models of primary neurodegeneration using FA values at the optic nerve

	***N***_***0***_	***r***_***0***_	***β***	**γ**	***R***^***2***^	***P***	***F-*test**
**Constant-rate model**							
Quantitative FA	0.64	0.0024	—	—	0.35	<0.05	—
Relative FA	—	0.0047	—	—	0.36	<1.0×10^-4^	—
**Risk-based model**							
*Constant-risk model*							
Quantitative FA	0.72	0.0081	—	—	0.67	<1.0×10^-3^	—
Relative FA	—	0.0084	—	—	0.89	<1.0×10^-5^	—
*Variable-risk model*							
Quantitative FA	0.73	0.0016	0.064	—	0.94	<1.0×10^-6^	*F*_*1,9*_ = 38.6*, *P* < 0.005
Relative FA	—	0.0019	0.057	—	0.98	<1.0×10^-7^	*F*_*1,10*_ = 37.9*, *P* < 0.0005
*Heterogeneity model*							
Quantitative FA	0.95	0.0031	0.060	0.58	0.96	<1.0×10^-7^	*F*_*1,8*_ = 3.5**, N.S.
Relative FA	—	0.0019	0.057	0.93	0.98	<1.0×10^-8^	*F*_*1,9*_ = 0.6**, N.S.

The quantitative and relative FA values were obtained from a region of interest in the glaucomatous optic nerve and from a ratio of FA between glaucomatous and control (contralateral) optic nerve, respectively. *F-test compared two nested models of constant-risk and variable-risk, taking into account the additional parameter *β* in the latter. **F-test compared two nested models of variable-risk and heterogeneity model, taking into account the additional parameter γ in the latter*.* N.S, not statistically significant.

The relevant statistics were also performed with voxel-based analysis of FA images, because this might help in finding a better model if it identified significant voxels located in known pathways of degeneration, such as the optic nerve or tracts. When both the cumulative risk and the post-operative period were entered as regressors in the statistical model, the FA images revealed a cluster located in the visual pathways, including the optic nerve and tracts, and the sagittal stratum, which were significant determinants in the coefficient of cumulative risk (Figure 1E, Additional file 2: Table S2), but not in the post-operative period. In addition, the decrease in FA was specific to the laser photocoagulation treatment; such a time- or risk-dependent decrease was not found in the FA values of the contralateral optic nerve (Additional file 3: Figure S1). Because the sagittal stratum includes the optic radiation, containing axons of transsynaptic neurons, we considered that the degree of secondary neurodegeneration could be also approximated as a linear function of the degree of primary neurodegeneration (see Appendix 1).

This was tested in detail by analysis with voxel-based regression and model selection. When voxel-based multi-regression analysis was performed using a regressor of the FA values at the primarily degenerated optic nerve, we found significant clusters for the contrast of primary-degeneration FA values at the bilateral optic radiation and the posterior callosum (Additional file 3: Figure S1C, Additional file 4: Table S3). These contain axons of the lateral geniculate and visual cortical neurons, respectively, which are known to be affected by transsynaptic neurodegeneration in glaucoma [16]. These results also depict the pathways to the superior colliculus, which is known to receive direct inputs from retinal neurons [35] (Additional file 3: Figure S1C, Additional file 4: Table S3). Transsynaptic neurodegeneration expressed by FA values at the optic radiation had a linear relationship with primary neuronal death as measured by FA at the optic nerve (*R*^*2*^ = 0.82, *P* < 0.0001, Additional file 5: Table S4), suggesting that this type of pathology has its basis in tight inter-neuronal interactions for cellular survival and supporting the hypothesized protective role of neurotropic factors in transsynaptic neurodegeneration [16]. The kinetics of FA values in the transsynaptic degeneration site were also an exponential function of the cumulative risk (*R*^*2*^ = 0.85 in ipsilateral and *R*^*2*^ = 0.80 in contralateral, Additional file 3: Figure S1E, Additional file 5: Table S4) and fitted by the variable-risk model significantly better than by the constant-risk model (*F*_*1,8*_ *=* 19.0, *P* < 0.005 in ipsilateral and *F*_*1,8*_ = 14.1, *P* < 0.01 in contralateral, Additional file 5: Table S4). Thus, these findings indicated that transsynaptic degeneration proceeds both in a linear relationship with the primary one, and in an exponential function of the cumulative risk.

**Figure 2:**
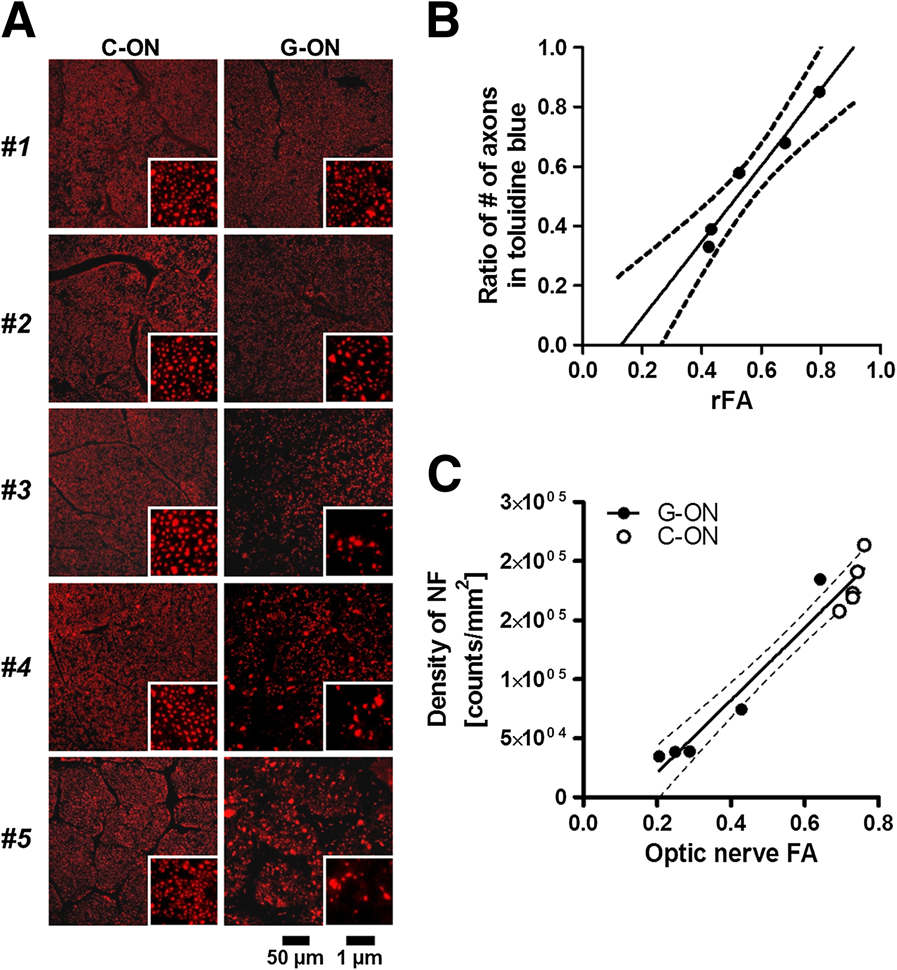
**The relationship between a DTI-based measures, FA, and a microscopic measurements, densities of axons.** (**A**) Sections of optic nerve immunohistochemically stained for phosphorylated neurofilaments (NF), in each side of the control (C-ON) and the glaucomatous optic nerve (G-ON), for each animal (#1–5). The density of NF was almost comparable to that of the contralateral control in the ocular hypertensive animal (#1), mildly decreased in the early stage glaucoma animal (#2), and severely degraded in advanced stage animals (#3–5). See Additional file 5: Table S5 for quantified data for each optic nerve. (**B**) A plot of FA values and the number of axons in the optic nerve. Values are ratios of the measured value in the glaucomatous side to that in the non-affected side. The numbers of axons were evaluated in transaxial sections of optic nerve stained with toluidine blue. (**C**) The relationship between DTI-based FA values (x-axis) and the microscopically-counted NF density (y-axis) in the glaucomatous (G-ON) and control optic nerve (C-ON). See also Additional file 5: Table S5 and Table 2.

Finally, we also tested whether the risk-based model could properly predict axonal degeneration. Axonal degeneration was measured by specific immunohistochemical staining with an SMI-31 antibody to evaluate phosphorylated neurofilament (NF), a marker of viable axons [36]. The NF density was better predicted by the variable-risk model than the constant-risk model by *F*-test (*F*_*1,3*_ = 29.7, *P* < 0.05, Table 2), strengthening the validity of the current model. Because our analysis has its basis in the assumption of linearity between imaging measures of FA and biological microstructures, such as axonal densities [31], this was also tested in our test animals with their optic nerves histologically analyzed. The ratio of optic nerve FA (glaucomatous/unaffected side) was correlated with that of axonal density (Figure 2B, Spearman’s *r* = 1.0, *P* < 0.05), which had been assessed in toluidine-blue stained sections. We also found that the FA values were significantly correlated with the density of NF-positive axons (Pearson’s *r* = 0.92, d.f. = 23, one-tailed *P* < 0.05, Figure 2C, Additional file 6: Table S5).

**Table 2:** Parameter estimates in the risk-based kinetic models of primary neurodegeneration using the L/R ratio of density of NF at the optic nerve

	***r***_***0***_	***β***	***R***^***2***^	***P***	*F-*test^*^
Constant-risk model	0.012	-	0.86	<0.01	-
Variable-risk model	0.0017	0.071	0.98	<0.05	*F*_*1,3*_ = 22.4, *P* < 0.05

^*^F-test compared the two nested models of constant-risk and variable-risk, taking into account the additional parameter *β* in the latter.

## Discussion

Using established experimental animals of neurodegeneration and *in vivo* neuroimaging data, we demonstrated the risk-dependent, stochastic nature of the kinetics of neurodegeneration, in which neurons probabilistically degenerate depending upon the product of time and amount of risk. Plausible exponential decay indicates that the earlier the disease risk is accumulated, the more rapidly neurodegeneration occurs, stressing the need for earlier detection using risk-related biomarkers. The current data showed that the risk-dependent decay spatially extended not only into the optic nerve but also into the remote white matter, consistent with the known widespread pathology of glaucoma in the visual pathways including the optic nerve, lamina cribrosa, lateral geniculate nucleus, and visual cortex [15,16]. The IOP increase has been known not only to elicit stress on the ganglion neurons and their unmyelinated axons in the retina or/and optic head, but also to induce glial activation and expression of tumor necrosis factor α, which may induce transsynaptic degeneration in remote areas [16,17]. The kinetics of the secondary transsynaptic neurodegeneration was approximated as a linear function of the kinetics of primary neurodegeneration, making the model effective when assessing degeneration in the brain with its enormous amount of inter-regional connectivity.

The current results provide three important findings as it relates to the kinetics of neurodegeneration. First, the results of our dynamic risk-based model for neurodegeneration confirm the stochastic nature of neurodegeneration in one-hit model, which was originally arisen from knowledge of inherited neurodegenerative diseases [11,37]. In the inherited trinucleotide repeat diseases, the age of symptomatic onset decreases exponentially with increasing length of trinucleotide repeats [37]. These diseases involve pathologies including initiation of nucleated polymerization, a rate-limiting thermodynamically unfavorable state, followed by rapid irreversible elongation to form fibrils [13,38]. Because the probability of the initial nucleation is increased by longer trinucleotide repeats, the risk function could simply be approximated as a step function across time; thus, the model explains the length-dependent variability in the age onset [13,37] and the exponential progress of neurodegeneration [11].

Second, the results extend the applicability of the risk-based stochastic model to the non-inherited, time or subject-variable neurodegenerative diseases. The variable-risk model better explained the variability of the data by 27% and 9% for quantitative and relative FA respectively (*R*^*2*^ = 0.94 and 0.98) than did the constant-risk model (*R*^*2*^ = 0.67 and 0.89, respectively). *F*-test for these nested models showed significant superiority of the variable-risk model (Table 1). This is important findings which indicate that the neurotoxic risk is not constant across time and subjects, even in typical animal model of neurodegeneration, which is all made by the same procedure. A majority of neurodegenerative diseases, such as Parkinson’s and Alzheimer’s diseases, do not involve trinucleotide repeats in the protein aggregation mechanisms and these diseases show significant between-subject variability in symptoms. This may be due to complicated pathomechanisms, such as the nonlinear nature of the risk [38,39], or multiple co-risks such as aging [9,40], microcirculation [41] and microglial activation [42]. The current result showing the close relationship between pathogenic (IOP) and neurodegenerative (DTI) biomarkers is also consistent with a recent finding in Alzheimer’s disease patients [43], whereby the deposition of beta-amyloid, a presumed pathogenic factor, was strongly related to hippocampal atrophy in the very early stage. Moreover, the current neurodegeneration model is virtually equivalent to those applied in the survival analysis of the Cox proportional-hazards regression model with time-dependent covariates (see Appendix 2). The model can further be generalized by including multiple risk factors (Appendix 2), such as genetic vulnerability (e.g. Apo E4 allele in Alzheimer’s disease), age, sex, and microcirculation, using multiplicative combination to form the net risk function (Eq. 8). The model can also be used for estimating the effect of neuroprotection by testing the interaction between the intervention and the risk to the time course of neurodegeneration.

Third, our findings of transsynaptic neurodegeneration suggest that the regionally exposed risk can induce system-wide neurodegenerative changes. The IOP increase may only injure the ganglion neurons in the retina, but the risk-based kinetic model allowed us to detect the FA decrease in the optic radiations, the 1st order transsynaptic sites. The observations are well consistent with findings by recent studies in glaucomatous patients [29,44]. The effect size may not be large in the higher order transsynaptic connections (as shown in Additional file 3: Figure S1); however, a specific spatial pattern covering the primary and secondary connections may increase diagnostic specificity for neurodegeneration that often proceeds in the specific neural circuit, e.g. visual pathways in case of glaucoma.

Although the heterogeneity model with stretched exponential decay (in Eq. 6) did fit well to our data, it did not significantly better explain our data than the risk-based model. The variability of the data was largely explained by the risk-based model (94-95%), which was improved only slightly by 1–2% when applied the heterogeneity model and F-test for model selection did not show significant difference between these two nested models (Table 1). Similarly, previous study showed that the stretched exponential model better fit to the survival curve of neurodegeneration using 16 kinds of data [33] and improved explanation of variability only by 0–11% (median = 4%), while the (constant-) risk-based model explained 72–98 % of data variability (median = 89%). Therefore, it is possible that the heterogeneity model may better explain the neuronal survival than the risk-based model, but the effect size seems to be relatively small as compared with models with less smaller number of parameters. Moreover, we consider that the model needs to be carefully assessed for its eligibility, particularly, for the parameter, γ. The value of γ may be influenced not only by biological factors (e.g. heterogeneity in the decay rate or late-stage biological compensatory response to the initial neurotoxic events) but also by measurement accuracy of the biomarker. Therefore, the actual factor that governs the variability of γ should be assessed systematically in each experimental condition. As for estimation of heterogeneity of decay rates, bi-exponential decay model could be better suited than the current model to evaluate the level of heterogeneity [45]. Moreover, since adding the parameter (γ) in the model may not only increase goodness of fit but also bias the base parameter (*β* or *r*_*0*_), it may need to be evaluated particularly when for example the study intervenes early-stage neuroprotection that may expect any change in *β*.

**Figure 3:**
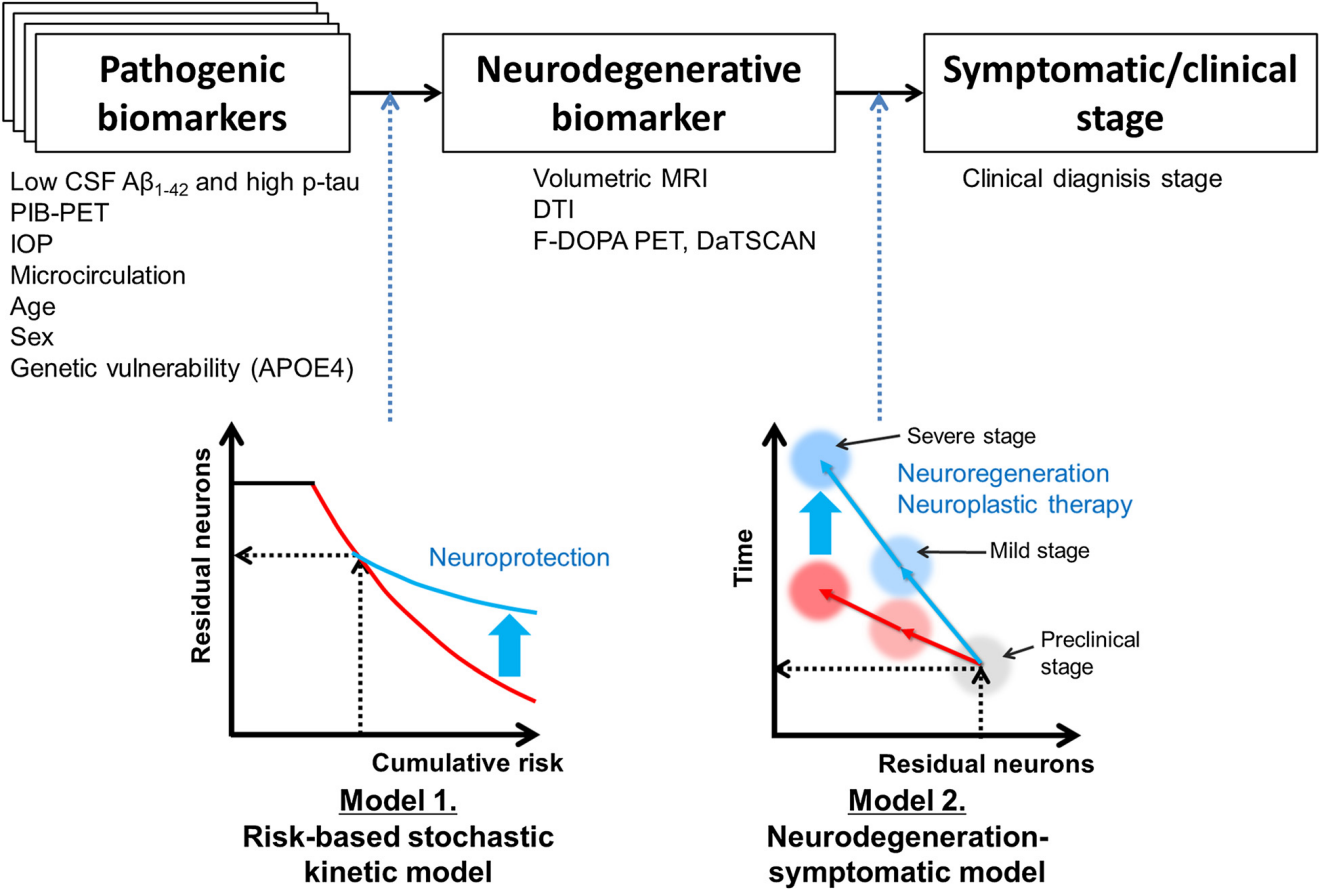
**A hypothetical model for biomarkers/clinical stages in neurodegenerative diseases.** The model involves a two-step causal pathway in the progression of neurodegenerative diseases: 1) a causal relationship of pathogenic biomarkers (PB) to a neurodegenerative biomarker (NB); and 2) a causal relationship of the NB to the symptomatic/clinical stages. As evidenced by the current study, the PB can interpret the kinetics of the NB as shown in the left lower graph (model 1, risk-based stochastic kinetic model). The heterogeneity in time for converting from a mild to a more severe clinical stage can be explained by the kinetics of the NB (model 2, neurodegeneration-symptomatic model). Optimal biomarkers for PB or NB may differ across different kinds of neurodegenerative diseases. For example, in glaucoma, the PB may be IOP and the NB may be DTI or structural MRI scans, as evidenced by the current study, whereas in Alzheimer’s disease [7], PBs may be low Aβ_1-42_ and high p-tau in cerebrospinal fluid (CSF), APOE genotype, PIB-PET and FDG-PET scans, while NBs may be structural MRI scans DTI findings; in Parkinson’s disease [8], PBs may be α-synuclein in CSF, sympathetic denervation in the heart (MIBG-SPECT), and LRRK2 gene mutation, while NBs may be F-DOPA PET and DaTSCAN (β-CIT) scans. From a therapeutic point of view, the earlier phase of the model may be the optimum time window for neuroprotection (blue arrow in left graph) and the later phase may be optimal for neuro-regeneration and neuroplastic therapies (blue arrow in right graph).

Based on the current results, we propose a two-step model for progression of neurodegenerative disease: a first step that correlates the “pathogenic” biomarker with the “neurodegenerative” biomarker, and a second one that correlates the neurodegenerative biomarker with clinical/symptomatic stage (Figure 3). This model may be similar to the recent biomarker model, where multiple biomarkers emerge in a cascade and are associated with the clinical stage [2], but it differs with respect to the emphasis on causality between cascades of biomarkers or clinical symptoms. In particular, the current risk-based stochastic kinetic model constitutes the first cascade between pathogenic and neurodegenerative biomarkers. Our approach may circumvent two problems related to biomarker accuracy. The first problem concerns the time lag between neurodegeneration and clinical manifestations. The current results show a kinetic relationship between pathogenic and neurodegenerative biomarkers, allowing prediction and therapeutic intervention to be made earlier, even in the preclinical stage. Thus, it is not necessary to do follow-up studies for a lengthy duration to await conversion from a preclinical (i.e., mild cognitive impairment, MCI) to a clinical stage (i.e. Alzheimer’s disease). In addition, inclusion of the time lag for clinical conversion may potentially have a problem of patient misclassification resulting from errors in clinical diagnosis. Modeling earlier disease stage is also important from a therapeutic point of view: the time lag between pathogenic and neurodegenerative biomarkers may be an optimal time window of neuroprotective therapies. The second problem that our approach circumvents concerns degraded predictability, which is inherent when using discrete variables (clinical stages) as an outcome. Our data showed that structural MRI data, which provides quantitative values, could be used as a neurodegeneration biomarker, as evidenced by its relationship to axonal densities. Besides the early stage, our two-step biomarker model assumes later-stage causality between neurodegeneration and clinical staging (Figure 3). This is consistent with knowledge obtained from functional localization in the brain and from the lesion studies in stroke and neurodegenerative diseases; thus heterogeneity in the neurological symptoms can be explained by variable regional distribution of the neurodegeneration. Therefore, rather than the pathogenic biomarker, the neurodegeneration biomarker may be predictive for symptomatic/clinical staging. This hypothesis was supported by a recent study that showed better performance of MRI volumetry of the hippocampus (as a surrogate of neurodegeneration) than amyloid retention in the brain (used as a pathogenic biomarker) for predicting the time of conversion from MCI to AD [46]. At this later stage, therapeutic intervention may include not only neuroprotection but also neurodegenerative and neuroplastic therapies (Figure 3).

A few issues suggest the need for some caution when applying the current approach. First, to increase generalizability, the current model needs to be tested in other kinds of neurodegenerative diseases and animal models. While the origin of glaucomatous pathology is chronic physical stress to retinal ganglion cells due to continuously increased intraocular pressure, most of neurodegenerative pathologies in Parkinson’ disease or Alzheimer’s disease is primarily based on the biochemical events, such as abnormal protein aggregations [47]. Therefore future studies should also address potential of the current model in various pathologies of neurodegeneration. Second, the predictability of the current model may depend on the measurements of pathogenic biomarker, such as initial time point or frequency. This effect is difficult to estimate without *a priori* knowledge of temporal variation in the pathogenic biomarker; thus pilot studies that estimate the potential variation may be required. In terms of diseases with abnormal protein aggregation, this effect may not be large because it takes years and even decades for the accumulation of proteins to occur. Third, the predictability of the model rests on the quality of biomarker data, and this may be different across diseases. For example, in Parkinson’s disease, fluorodopa PET can be used as a neurodegeneration biomarker because the uptake constant of fluorodopa has a direct linear relationship to the number of surviving dopaminergic neurons in the substantia nigra [48]. A potent pathogenic role of microglial activation can be estimated by testing the predictability of the model using PET and ^11^C-PK11195, a marker of translocator protein [49,50].

## Conclusions

In summary, we have successfully explained the kinetics of glaucomatous neurodegeneration by a dynamic risk-based kinetic model and *in vivo* measurement of biomarkers. The results support that neurodegeneration occurs and proceeds as a probabilistic events based on the risk to neurons. Although many studies may be preferable in other kinds of neurodegenerative diseases to generalize the current kinetic model, the model may provide opportunities for predicting the time course of neurodegeneration in the living brain in the early stage of the disease, and may bridge the dissociation between heterogenous pathology in patients and standardized pathology in experimental animal models of neurodegeneration [51].

## Methods

### Kinetic models of neurodegeneration

In the risk-based stochastic model, the rate of decrease in neuronal/axonal density is a product of the risk of cell death at time *t*, *r(t),* and the density of neurons/axons, *N(t),* which can be expressed as follows,

(1) $$\mathrm{dN}\left(t\right)/\mathrm{dt}=-r\left(t\right)\cdot N\left(t\right)$$

The *r(t)* may be thought of as the instantaneous probability of cell death at a particular time, *t*. Solving differential equations of Eq. 1 generates the following equation:

(2) $$N\left(t\right)=N_{0}\exp\left(-\int r\left(t\right)\;\mathrm{dt}\right)$$

where *N*_*0*_ is the number of neurons at time zero. The function for risk, *r(t),* should be non-negative and could be substituted by an exponential function as follows:

time-variable risk:

(3) $$r\left(t\right)=r_{0}\cdot \exp\left(\beta \cdot p\left(t\right)\right)$$

or

time-invariable (constant) risk:

(4) $$r\left(t\right)=r_{0}$$

where *r*_*0*_ constitutes a component of baseline risk, while the other term, exp(*β · p(t)*)*,* constitutes the risk. In the variable-risk model (Eq. 3), the risk for glaucoma, intraocular pressure (IOP), was incorporated into the *p(t),* which was calculated as measured IOP values minus 29 mmHg, the 95% confidence upper limit of IOP in the normal optic nerve. The optimal values for *r*_*0*_*, β*_*,*_*N*_*0*_ were estimated to obtain the best fit of the model by a non-linear least square method using the values of IOP and FA in the glaucomatous optic nerves at each time point.

For the constant-risk model, *β* in Eq. 3 was fixed to zero, thus forming Eq. 4, which corresponds to that used in previous literature (Eq. 3 in ref*.*[11]), and the values of *r*_*0*_ and *N*_*0*_ were optimized in the fitting procedure. Besides these two risk-based models, we also evaluated the classical model (constant-rate model) of neurodegeneration [10], in which neuronal loss proceeds at a constant rate, as follows:

(5) $$\mathrm{dN}\left(t\right)/\mathrm{dt}=-r_{0}$$

where *r*_*0*_ is a rate constant for the decrease in the number of neurons, expressed as counts per unit of time. The initial condition was again substituted by *N(0) = N*_*0*_.

In addition, we considered a similar but another model of neurodegeneration kinetics, recently proposed for heterogeneous kinetics [33]. The model has been often used in the field of physics or engineer to describe the discharge of capacitors, relaxation of disordered systems or luminescence decay [34], and is recently applied to several biological data such as estimation of human lifespan [52] and kinetics of heterogeneous neurodegeneration [33].

(6) $$N\left(t\right)=N_{0}\exp\left(-{\left(\int r\left(t\right)\mathrm{dt}\right)}^{\gamma }\right)$$

In this model, γ is a shape parameter such that if 0 < γ < 1, *N(t)* shows a typical form of stretched exponential decay across ∫ *r*(*t*)*dt*, while if γ = 1 it becomes identical to the original risk-based exponential decay as in Eq. 2, and if γ > 1 sigmoidal (or compressed exponential) decay. Mathematically, the function is also known as a Weibull function [53].

### Animals

Eight macaque monkeys (*Macaca fascicularis*, body weight 4–5 kg, all male) were used; five animals were used to model glaucomatous pathologies, while three were control. The animal model of glaucoma was made using an established method of laser photocoagulation, as described previously [20]. Glaucoma model animals were evaluated in detail for histological changes in microglial cells, astrocytes, and neurons in the lateral geniculate nucleus, and the results were published elsewhere [50]. Visual field defects and pathological features of this animal model are also described in detail in our previous studies [19,20,54]. Before being enrolled into these experiments, all animals were confirmed by ophthalmoscopy to have no abnormalities in their ocular fundus. After glaucoma was induced, the animals were repeatedly followed up with measurements of intraocular pressure (IOP) and diffusion tensor imaging. All surgical and experimental procedures conformed to NIH guidelines for the care of experimental animals (National Institutes of Health Committee on Care and Use of Laboratory Animals, 1985). The study was approved by the Institutional Animal Care and Use Committee of RIKEN and the National Cardiovascular Center Research Institute.

### Induction of experimental glaucoma

Elevated IOP was induced by applying argon blue/green laser photocoagulation burns to the trabecular meshwork of the left eye, with the right eye being used as an untreated control. For the laser treatment, the animals were anesthetized with an intramuscular injection of ketamine (8.75 mg/kg) plus xylazine (0.5 mg/kg). A single-mirror Goldmann lens filled with a physiological solution was placed on the eye to be treated. The argon laser was focused on the mid-portion of the trabecular meshwork, and a total of 150 laser-beam spots were applied around 360° (spot size 100 μm; power 1.0 W; exposure time 0.2 sec) using an argon laser photo-coagulator (Ultima 2000 SE; Coherent Inc., CA, USA) attached to a standard slit-lamp microscope (BQ 900; Haag-Streit, Köniz, Switzerland). The same laser treatment was repeatedly applied two weeks after the first treatment to maintain continuous IOP elevation, as has been confirmed previously. Potential complications of the glaucoma surgery, including retinal ischemia were not suspected by detailed histological evaluations [50].

### IOP

The IOP was intermittently measured (over an interval of 3–28 days) in both eyes during the course of glaucoma in each animal using a calibrated applanation pneumotonometer (Model 30 Classic Pneumotonometer; Medtronic Solan, FL, USA). The measurement was performed under generalized anesthesia using intramuscular ketamine (8.75–10 mg/kg) and local anesthesia with 0.4% oxybuprocaine hydrochloride.

### DTI

Diffusion tensor magnetic resonance images (DTI) were acquired using a 3-Tesla magnetic resonance image (MRI) scanner (Signa Horizon Lx VH3, General Electric Healthcare, Little Chalfont, Buckinghamshire, UK), which provides a maximum gradient strength of 40 mT m^-1^, rising in 268 μs using a customized eight-channel phased array receiver coil. During the DTI scanning, animals were intubated and deeply anesthetized using a gaseous anesthetic, 1.5% isoflurane, with their respiration assisted by a ventilator (Cato, Dräger, Germany). To maintain stable physiological conditions, we monitored the partial pressures of oxygen, carbon dioxide and isoflurane in the inspiratory and expiratory gases. We also intermittently performed gas analysis of the arterial blood that was collected from the tail artery, in which a 24-gauge needle was indwelled. The animal’s head was fixated to a customized acrylic retainer that was firmly attached to the receiver coil. The animal and the retainer were placed on the MRI gantry with the animal’s head centered in the bore of MRI scanner. After scanning a localizer for planning a field of view for subsequent scans, a higher-order shimming was performed using a spiral sequence that allows us to calculate the fitted field map including second-order components (for a total of 10 components), and to minimize inhomogeneity in the static magnetic field. Then, the DTI data were collected using a multi-shot spin-echo type EPI sequence (number of shot = 2, TR = 17000 ms, TE = 81.9 ms, FA = 90°, number of slice = 51) with an isotropic spatial resolution of 0.9 mm and with diffusion-weighted gradients of 27 directions (b value = 1000 s mm^-2^). The diffusion-weighted gradients were applied in a symmetrical, twice-refocused pulse to reduce eddy-current-induced distortion in the DTI images [55]. The DTI data, a total of 24 volumes for three non-diffusion-weighted volumes and 21 diffusion-weighted volumes, were obtained by three separate scans, each consisting of one non-diffusion and seven diffusion-weighted volumes. Before each scan, we monitored the temperature of the gradient coil, and started the scan when the temperature was between 22° and 23°C, at which the shift and distortion across scans were minimized. If the temperature was less than that specified, we performed dummy scans to keep the temperature within range. The DTI data were scanned three times for each time point during the follow-up of glaucoma. The total time for obtaining DTI data was approximately 5 hours, depending on the temperature and computation time for the image reconstruction. We also scanned two gradient-echo sequences with different TE values (TR = 200 ms, TE1 = 4.4 ms, TE2 = 6.637 ms) and obtained the field map to be used for a post-process of reducing distortion of DTI images, which originates from the magnetic field inhomogeneities caused by magnetic susceptibility differences between neighboring tissues, such as air-bone or air-tissue.

### Image analysis

Raw DTI data were corrected for image shift, rotation and distortion (with a linear transformation of 12 degrees of freedom) and for B0-inhomogeneity distortion (with non-linear warping calculated on the fieldmap) using the programs FLIRT and PRELUDE, respectively, both part of the Functional magnetic resonance imaging of the brain (FMRIB) Software Library (FSL) [56], developed by the Analysis Group of FMRIB Centre, University of Oxford. Estimated shifts for these corrections were concurrently applied to the original images to minimize reslicing noise in the corrected data. Then, the image for fractional anisotropy (FA) was computed by fitting a diffusion tensor model [30] to the corrected diffusion data using the program, FDT, part of the FSL.

For region of interest (ROI) analysis of FA based on the primary degeneration kinetic model, we delineated the ROI with a columnar shape (3 mm long, diameter of 2 mm, number of voxels of 12), centered at the center of the optic nerve, at a distance of 6 mm from the junction of the eyeball and the nerve. For each ROI, we obtained FA values averaged across three scans obtained at the same time point. For fitting the kinetic models of primary neurodegeneration, we used a quantitative value of FA or ratio of FA (*rFA*, expressed as a ratio to the FA of the contralateral optic nerve) in glaucomatous animals and determined optimum values for *N*_*0*_, *r*_*0*_ and *β*. When we used *rFA*, *N*_*0*_ was fixed to one. The integration of risk function, *r(t)*, in Eq. 1 was calculated based on the trapezoid rule using discrete time point data for IOP. The goodness of fit was evaluated based on coefficients of determination (*R*^*2*^) and the *P*-value computed in the fitting process. The *F*-test for model selection was performed for the two nested models between with constant- and variable-risk or between with variable-risk and heterogeneity. The fitting and *F*-test were computed using Pybld (http://www.mi.med.osaka-u.ac.jp/pybld/pybld.html) built in the language Python. We also confirmed that the goodness of fits in the stochastic variable-risk model was independent of the arbitrarily determined threshold (29 mmHg, 95% confidence upper limit of normal IOP). For presenting graphs, the optimized values determined at the fitting process for FA data, was used for *N*_*0*_, *r*_*0*_ and *β* to calculate the cumulative risk, ∫exp(*r(t))dt* to be used in the x-axis of graphs (Figure 1D, 1F and Additional file 3: Figure S1E) and for making the regressor in the subsequent analysis of voxel-based statistics and of secondary neurodegeneration (see below). FA and rFA values in normal animals were used for the scatter plot, but not for fitting the kinetic models of neurodegeneration. For graph plotting, the cumulative risks of control (contralateral to glaucomatous side) and normal optic nerves were considered to be zero, which means that baseline risk, *r*_*0*_, was also zero.

For voxel-based analysis of FA images, we used tract-based spatial statistics (TBSS) [57], part of the FSL. First, FA images were brain-extracted using BET [58] and were aligned into a common space (a matrix of 137 × 167 × 100; a voxel size of 0.4 mm-cubic) using the nonlinear registration tool FNIRT, part of the FSL, which uses a b-spline representation of the registration warp field [59]*,* followed by linear registration to the standard space of the anterior-posterior commissure line of the macaque brain [60]. Next, the mean FA image was created and thinned to create a mean FA skeleton representing the centers of all tracts common to all data. The mean FA skeleton was created by thresholding the mean FA image at an FA value larger than 0.3. Each scan’s aligned FA data were then projected onto this skeleton and the resulting data fed into voxel-wise cross-subjects statistics for the linear regression analysis. The statistical analysis for estimation of the neurodegenerative model included analyzing the skeletonized FA images by testing significance of coefficients of post-operative time and the cumulative risk in the multi-regression statistical model. The analysis for the secondary neurodegenerative changes involved analyzing the skeletonized FA images by testing regressors of FA values in the affected optic nerve and of post-operative time. The predictor in the variable-risk model was made by using the optimized values for *N*_*0*_, *r*_*0*_ and *β* determined from prior analysis of the quantitative FA in the primary degenerated area, the optic nerve (Table 1). For the statistical threshold, we applied threshold-free cluster-corrected *P*-values less than 0.05. Voxel-wise statistics were performed by permutation-based nonparametric inference using the program Randomise, part of the FSL. For post hoc analysis of the model of secondary neurodegeneration, a cubic ROI consisting of 27 voxels was placed with the center of the ROI located at the local maximum in the optic radiation in the contrast for the regressor of optic nerve FA. The model equation, Eq. 6, was fitted to the FA values in the areas of primary degeneration (glaucomatous optic nerve) and secondary degeneration (optic radiation), and the optimum values for *k*_*t*_ and *N*_*B0*_ were determined. The model was also built based on the risk, by substituting *N*_*A*_*(t)* in Eq. 6 by Eq. 2, then was fitted to the FA data of the optic radiation to determine optimum values for *N*_*0*_, *r*_*0*_, and *N*_*B0*_. An *F*-test was performed for model selection between constant- and variable-risk models, taking into account the additional parameter (*β*) in the latter.

### Histology

The animals were analyzed by conventional histological evaluations, including toluidine blue and cresyl violet staining of retina, optic nerve and lateral geniculate nucleus. In addition, to correlate the optic nerve DTI changes with the degeneration of axons, optic nerve immunofluorescence staining was performed using SMI-31 antibody, a maker of neurofilament (NF), a constituent of neuronal axons. Animals were euthanized 33–168 days after glaucoma induction under deep general anesthesia (isoflurane in N_2_O and O_2_ by inhalation) and were perfused via the common carotid artery with 1 L of 0.9% saline containing 10 U/ml heparin at room temperature, followed by 1 L of 4% paraformaldehyde in 0.01 M phosphate-buffered saline (PBS; pH 7.4). Eyeballs with optic nerves were enucleated at the time of brain removal and 4% paraformaldehyde in PBS solution was injected into the vitreous body and postfixed by immersion in 4% paraformaldehyde in PBS for at least 1 week at 4°C. After immersion, a 3-mm long segment of optic nerve was cut out at 6 to 9 mm from the eyeball–optic nerve junction. The eyeballs and optic nerve segments were soaked in 10, 15, and 30% (w/v) sucrose in 0.1 M phosphate buffer, pH 7.4, at 4°C, for at least 24 h each, and then frozen in embedding compound (Tissue-Tek; Sakura Finetechnical Co.Ltd., Tokyo, Japan).

Coronal sections of the optic nerve segment were cut at 10-μm thickness and every 20th section was mounted onto the same slide glass until we obtained 20 slide glasses each containing four sections. Because cutting of the sections started from the end closer to the eyeball, the sections evaluated for counting should be 6–7 mm distant from the eyeball–nerve junction. Optic nerve sections were washed with 0.01 M PBS, preincubated with 10% normal goat serum in 0.01 M PBS for 30 min, and then incubated overnight at 4°C with mouse anti-SMI31 monoclonal antibody (NE1022; 1: 1000, Calbiochem, San Diego, CA, USA). They were washed with 0.01 M PBS and then incubated for 3 h at room temperature with Alexa Fluor 546 F(ab’)2 fragment of goat anti-mouse IgG (H + L) (1:1000 dilution) (Molecular Probes, Eugene, OR). For counting the number of SMI-31-positive axons, we randomly chose a single slide glass from the twenty, and identified five regions (nasal, temporal, superior, inferior, and central) in each of the four optic nerve sections. In the center of each region, we placed a sample volume of interest with a size of 218 × 164 × 10 μm (in x, y, and section thickness) under a microscope fitted with a 40× objective × 3 digital zoom; thus, a total of 0.18 mm^2^ per section (areal ratio of 3.6 ± 0.4 % as mean ± S.E.) was assessed for counting the number of SMI-31-positive (i.e. NF-positive) axons. Counting and photography were performed by a single observer who was blinded to the animals’ data and DTI results (Y. I). Thereby, for each optic nerve, we obtained 20 measurements of axon densities (five regions × four sections) and calculated the mean value that represents the NF-positive axon density of the corresponding optic nerve.

The obtained NF-positive axon density was used as an independent variable in the linear regression analysis of optic nerve FA. The ratio of NF density (between the glaucomatous and contralateral optic nerve) was used for fitting the risk-based kinetic models of neurodegeneration and determining the optimum values for *r*_*0,*_ and *β.* Model selection was also analyzed using an *F*-test between constant- and variable-risk models. Although the absolute NF density in monkey #1 (mild stage glaucomatous animal) was almost comparable with those in normal nerves (Additional file 6: Table S5), the ratio (to that in the contralateral non-affected optic nerve) was slightly decreased (0.86), similar to the axonal density (0.85) as evaluated by toluidine blue staining. In fact, toluidine blue-stained sections of this animal’s affected optic nerve revealed neurodegeneration and formation of myelin ovoids in the peripheral part of the optic nerve.

## Appendix 1

### Model for secondary (transsynaptic) neurodegeneration

We considered a case in which region A suffers from primary neurodegeneration due to primary neurotoxic risk, while a remote area, region B, which is connected to region A, suffers from the risk of transneuronal neurodegeneration due to the primary degeneration in region A. The kinetics of secondary neurodegeneration in region B (*N*_*B*_*(t)*) was assumed to have a linear relationship with that of the primary neurodegeneration in region A (*N*_*A*_*(t)*) as follows,

(7) $$\frac{dN_{B}\left(t\right)}{\mathrm{dt}}=k_{t}.\frac{dN_{A}\left(t\right)}{\mathrm{dt}}$$

where *k*_*t*_ is a constant coefficient. The value of *k*_*t*_ should depend both on the strength of the trans-synaptic neurodegeneration and the connectivity between regions A and B, which may vary from region to region, or connectivity strength to the primary lesion in the brain. The differential equation (Eq. 7) indicates that the number of neurons in the area B (*D*_*B*_*(t)*) should decrease as a linear function of the primary degenerated area A (*D*_*A*_*(t)*) (the intercept of this linear function was substituted by *N*_*B0*_) as illustrated in Additional file 3: Figure S1A. Thus, if the neurodegeneration kinetics of the latter region is an exponential function of the cumulative risk (Eq. 2), the kinetics in region B should also be a decremental exponential function across the cumulative risk, but approach the plateau, *N*_*B0*_, at infinite time (Additional file 3: Figure S1B).

## Appendix 2

### General risk-based kinetic model of neurodegeneration

As an extension of the time-variable risk model, we also considered the situation where multiple factors are involved in the risk of neurodegeneration. If these multiple risk factors expose one to neurodegenerative risk independently, the net risk is supposed to be a multiplication of the risk function of each risk factor. Thus, the net risk function in Eq. 3 can be generalized into the following equation:

(8) $$r\left(t\right)=r_{0}.\exp\left(\beta_{1}.p_{1}\left(t\right)+\beta_{2}.p_{2}\left(t\right)+\cdot \cdot \cdot +\beta_{n}.p_{n}\left(t\right)\right)$$

The general form of neurodegeneration kinetics, as formulated in Eq. 2 and 7, is virtually equivalent to those used in the survival analysis of the Cox hazard proportional regression model with time-dependent covariates [61,62]. Thus, the treatment effect could be estimated by testing the significance of a covariate for the treatment or an interaction term between the pathogenic biomarker and the treatment.

## Competing interests

There are no non-financial competing interests to declare in relation to this manuscript.

## Authors’ contributions

TH carried out the MRI experiments, analysis and wrote the manuscript. MS carried out the preparation of animal model and helped to draft the manuscript. HW wrote the program for kinetic model. TO carried out the MRI experiment. YI carried out IOP measurements. HY carried out MRI experiment. SU wrote the MRI sequence program. HW participated in the design of the study. HH participated in the design of the stuty. HO participated in the design of the study and coordination. All authors read and approved the final manuscript.

## Supplementary Material

Additional file 1: Table S1 List of animals with monocular glaucomatous neurodegeneration. Click here for file

Additional file 2: Table S2 Significant FA changes and their correlation with cumulative risk. Click here for file

Additional file 3: Figure S1 Transsynaptic secondary neurodegeneration – kinetic model and findings in glaucomatous animals. Click here for file

Additional file 4: Table S3 Significant FA changes and their correlation with FA values for the glaucomatous optic nerve. Click here for file

Additional file 5: Table S4 Parameter estimates in the kinetic models of secondary neurodegeneration using FA values at the optic radiation (OR). Click here for file

Additional file 6: Table S5 Quantitative results of immunostained phosphorylated neurofilaments (NF) in the optic nerve in monocular glaucomatous model animals. Click here for file
