# Supplementary material for: Kinetics of neurodegeneration based on a risk-related biomarker in animal model of glaucoma
Source: Mol Neurodegener. 2013 Jan 18;8:4. doi: 10.1186/1750-1326-8-4 (PMC3599096; doi:10.1186/1750-1326-8-4)
Supplement: Additional file 1: Table S1 — List of animals with monocular glaucomatous neurodegeneration. [file 1750-1326-8-4-S1.pdf]

Table S1. Animals with monocular glaucomatous neurodegeneration

Animal ID	Duration of observation	IOP (mmHg)				Ophthalmoscopic findings			
	(days)	Baseline		Mean	Maximal	Baseline C/D		Maximal C/D	Glaucomatous stage*
		R	L	L	L	R	L	L	L
#1	65	22.6	24.3	40.2	52.7	0.15	0.19	0.20	Mild
#2	33	21.7	23.4	61.3	72.0	0.26	0.25	0.40	Moderate
#3	78	21.0	20.3	60.2	73.3	0.22	0.17	0.64	Advanced
#4	106	24.3	24.7	62.1	70.7	0.18	0.19	0.56	Advanced
#5	168	24.8	22.4	50.8	60.0	0.23	0.23	0.77	Advanced

IOP, intraocular pressure; C/D, cup to disc area ratio; L, left; R, right. *Glaucomatous stage was diagnosed by an experienced observer based on ophthalmoscopic findings.
