# Supplementary material for: Kinetics of neurodegeneration based on a risk-related biomarker in animal model of glaucoma
Source: Mol Neurodegener. 2013 Jan 18;8:4. doi: 10.1186/1750-1326-8-4 (PMC3599096; doi:10.1186/1750-1326-8-4)
Supplement: Additional file 2: Table S2 — Significant FA changes and their correlation with cumulative risk. [file 1750-1326-8-4-S2.pdf]

Table S2. Significant FA changes and their correlation with cumulative risk

Regressors	Regions	X	Y	Z	T_{max}
Cumulative risk	Optic nerve	-4	6.4	-6	7.4
	Optic tract	6.8	-4.8	-6	7.3
		-6	-3.2	-6	6.4
	Sagittal stratum (Optic radiation)	10.4	-34.4	-0.8	5.9
		-12	-33.6	-0.6	5.8
Post-operative time	None				

The table lists regions with a significant effect (cluster-corrected P<0.05).
