# Supplementary material for: Kinetics of neurodegeneration based on a risk-related biomarker in animal model of glaucoma
Source: Mol Neurodegener. 2013 Jan 18;8:4. doi: 10.1186/1750-1326-8-4 (PMC3599096; doi:10.1186/1750-1326-8-4)
Supplement: Additional file 3: Figure S1 — Transsynaptic secondary neurodegeneration – kinetic model and findings in glaucomatous animals. [file 1750-1326-8-4-S3.pdf]

Figure S1

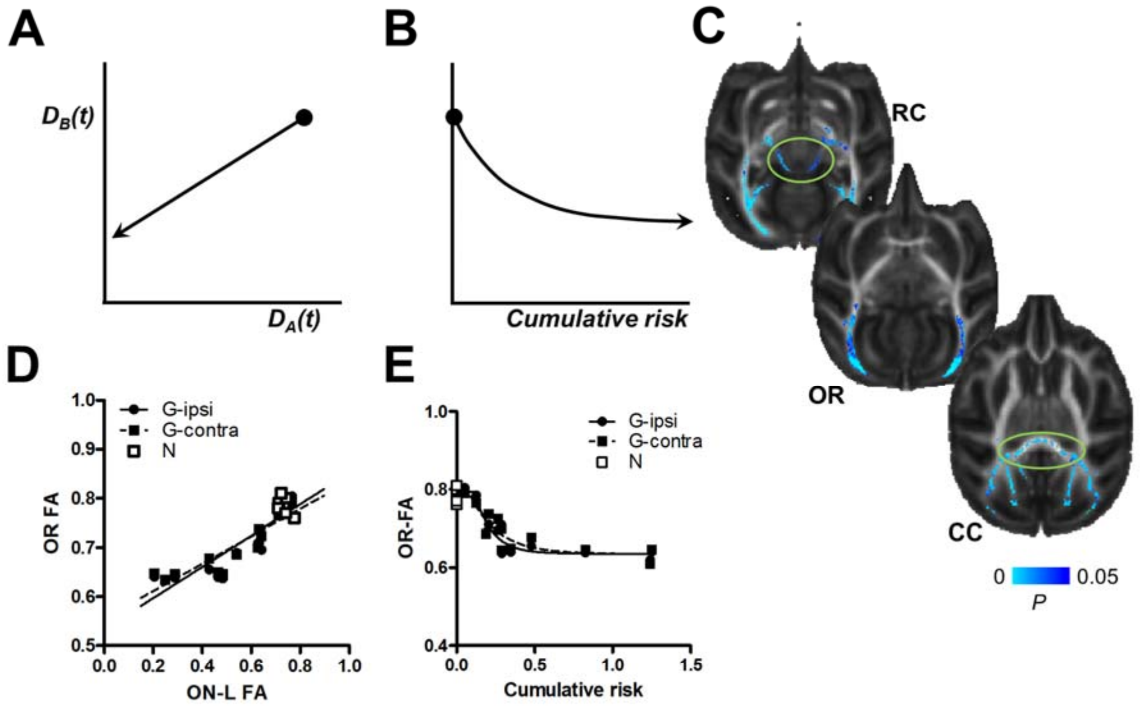

Fig. S1. Transsynaptic secondary neurodegeneration – kinetic model and findings in glaucomatous animals.

(A) A relationship of neurodegeneration in region A (primary neurodegeneration area) and B (transsynaptically-connected area), as predicted by the kinetic model of transsynaptic neurodegeneration.

(B) Neurodegeneration in region B (y-axis) plotted across cumulative risk (x-axis), predicted by the linear model of transsynaptic neurodegeneration.

(C) The results of voxel-based linear regression analysis of FA images using FA values in the glaucomatous side (left eye) of the optic nerve as a regressor. RC, reticulo-collicular tracts; OR, optic radiation; CC, corpus callosum. See Table S3 for all the lists of significant regions.

(D) A plot of FA values of both sides of the optic radiation, the secondary neurodegeneration areas (y-axis) across those of glaucomatous optic nerve, the primary neurodegeneration area (x-axis). The FA values of optic radiation was obtained from the region of interest centered at x, y, z = -12, 34.4, 0.4 mm (ipsilateral, G-ipsi) and 10.4, -34, 0.4 mm (contralateral, G-contra) in glaucomatous animals. For comparison, the values in normal animals (N) were also plotted.

(E) A plot of the FA values of both sides of the optic radiation (y-axis) across the cumulative risk (x-axis). Parameter estimates are listed in Table S4.
