# Supplementary material for: Kinetics of neurodegeneration based on a risk-related biomarker in animal model of glaucoma
Source: Mol Neurodegener. 2013 Jan 18;8:4. doi: 10.1186/1750-1326-8-4 (PMC3599096; doi:10.1186/1750-1326-8-4)
Supplement: Additional file 4: Table S3 — Significant FA changes and their correlation with FA values for the glaucomatous optic nerve. [file 1750-1326-8-4-S4.pdf]

**Table S3**. Significant FA changes and their correlation with FA values for the glaucomatous optic nerve

| Regressors | Regions | X | Y | Z | $T_{max}$ |
|---|---|---|---|---|---|
| Optic nerve FA | Optic nerve | -4 | 5.6 | -6.4 | 10.2 |
| | Optic tract | -6.4 | -4 | -6.4 | 8.3 |
| | | 7.2 | -5.2 | -6.4 | 7.6 |
| | Reticulo-collicular pathway | 4.4 | -15.6 | -2 | 6.8 |
| | Sagittal Stratum (Optic radiation) | 10.4 | -34 | 0.4 | 7.2 |
| | | -12 | -34.4 | 0.4 | 8.2 |
| | Posterior callosum | -2.8 | -16 | 4.8 | 7.1 |
| Post-operative time | None | | | | |

The table lists regions with a significant effect (cluster-corrected P<0.05). Optic nerve FA was obtained from the glaucomatous side.
