# Supplementary material for: Kinetics of neurodegeneration based on a risk-related biomarker in animal model of glaucoma
Source: Mol Neurodegener. 2013 Jan 18;8:4. doi: 10.1186/1750-1326-8-4 (PMC3599096; doi:10.1186/1750-1326-8-4)
Supplement: Additional file 5: Table S4 — Parameter estimates in the kinetic models of secondary neurodegeneration using FA values at the optic radiation (OR). [file 1750-1326-8-4-S5.pdf]

Table S4. Parameter estimates in the kinetic models of secondary neurodegeneration using FA values at the optic radiation (OR)

**Model for secondary neurodegeneration based on risk**					
	N_0	r_0	N_{B0}	R^2	*F*-test*
Constant-risk model					
L OR	0.89	0.037	0.63	0.40	-
R OR	0.78	0.021	0.63	0.31	-
Variable-risk model					
L OR	0.80	0.0061	0.63	0.86	$F_{1,8}$=19.0, P<0.005
R OR	0.78	0.0040	0.63	0.82	$F_{1,8}$=14.1, P<0.01

**Linear model between primary and secondary neurodegeneration**				
	k_t	N_{B0}	R^2	P
L OR	0.34	0.52	0.82	<0.0001
R OR	0.36	0.52	0.82	<0.0001

The quantitative values of FA were obtained from regions of interest (ROIs) in the optic radiation, centered at x, y, z = 10.4, -34, 0.4 mm (left) and –12, 34.4, 0.4 mm (right) in glaucomatous animals. In the variable-risk model, the value of β was substituted by 0.064, which was determined from the variable-risk model and quantitative FA at primary degenerated area, optic nerve (see Table 1). *F-test comparing the two models with constant-risk and variable-risk, the latter taking into account the additional parameter β.
