# Supplementary material for: Kinetics of neurodegeneration based on a risk-related biomarker in animal model of glaucoma
Source: Mol Neurodegener. 2013 Jan 18;8:4. doi: 10.1186/1750-1326-8-4 (PMC3599096; doi:10.1186/1750-1326-8-4)
Supplement: Additional file 6: Table S5 — Quantitative results of immunostained phosphorylated neurofilaments (NF) in the optic nerve in monocular glaucomatous model animals. [file 1750-1326-8-4-S6.pdf]

Table S5. Quantitative results of immunostained phosphorylated neurofilaments (NF) in the optic nerve in monocular glaucomatous model animals

Animal ID	Density of NF in optic nerve		
	R (control) (counts/mm^2)	L (glaucoma) (counts/mm^2)	L/R ratio
#1	213706	184567	0.86
#2	157555	74521	0.47
#3	169081	38889	0.23
#4	172744	38523	0.22
#5	190840	34696	0.18

NF, phosphorylated neurofilament; R, right; L, left.
